# Differentiating Fetal Alcohol Spectrum Disorder from other neurodevelopmental disorders: neurocognitive and socio-emotional evidence

**DOI:** 10.3389/fnins.2025.1716494

**Published:** 2025-12-05

**Authors:** Roberto Fernandes-Magalhaes, Lorena Labrado, David Ferrera, Marisa Fernández-Sánchez, Ana Belén del Pino, Dino Soldic, Sarai Pazos-García, María Eugenia De Lahoz, Irene Peláez, Paloma Barjola, Francisco Mercado

**Affiliations:** 1 Department of Psychology, Faculty of Health Sciences, Rey Juan Carlos University, Madrid, Spain; 2 Research Group on Cognitive Neuroscience, Pain and Rehabilitation (NECODOR), Rey Juan Carlos University, Madrid, Spain

**Keywords:** Fetal Alcohol Spectrum Disorder, neurodevelopmental disorders, cognitive impairments, behavior, neuropsychology

## Abstract

**Background:**

Fetal Alcohol Spectrum Disorder (FASD) is associated with widespread cognitive, behavioral, and adaptive impairments. Its clinical presentation often overlaps with other neurodevelopmental conditions (ND), such as learning disorders (LD) or Attention-Deficit/Hyperactivity Disorder (ADHD), making differential diagnosis challenging. Although syndrome-specific cognitive patterns have been suggested, a distinctive neurocognitive profile of FASD remains inconclusive.

**Method:**

Seventy-six children (46 with FASD and 30 with ND), aged 6–15 years (*M* = 11.00, SD = 2.30), completed a comprehensive neuropsychological battery, covering domains of intellectual functioning, attention, memory, verbal skills, executive functions, and socio-emotional behavior.

**Results:**

As compared to normative ranges, children with FASD felt below expected levels in cognitive functioning. Moreover, children with FASD showed significantly lower performance than those with ND across all cognitive domains, including global intelligence, verbal comprehension, working memory, processing speed, attention, memory, and executive functions. At the socio-emotional level, parental reports revealed that children with FASD scored higher than their peers with ND on anxiety, social and thought problems, attentional difficulties, and aggression behavior, although most of them remained within non-clinical ranges.

**Conclusion:**

Findings support the presence of a distinctive neurocognitive profile in FASD characterized by generalized cognitive deficits and domain-specific impairments (particularly in attention, memory, and executive functions). In addition, children with FASD scored higher on socio-emotional difficulties, although still within non-clinical ranges. These results highlight the clinical relevance of domain-specific assessment and underscore the need for early diagnosis and targeted evidence-based interventions to mitigate long-term cognitive and adaptive challenges associated with prenatal alcohol exposure.

## 1 Introduction

Fetal Alcohol Spectrum Disorder (FASD), in any of its subtypes (Hoyme et al., 2016), is the clinical term that encompasses a set of neurological and clinical signs observed in individuals who were exposed to alcohol during the gestational period (Wozniak et al., 2019). Among the effects of prenatal alcohol exposure, scientific evidence has highlighted the presence of central nervous system alterations affecting the child’s normal development involving not only the physical, but also cognitive, emotional, and behavioral spheres (Nuñez et al., 2011; Popova et al., 2023; Rockhold et al., 2023; Roebuck et al., 1998). Physically, individuals with FASD often present alterations in height and weight (below 10 percentile) (Caputo et al., 2016; Carter et al., 2016), as well as craniofacial malformations (e.g., microcephaly) (Petrelli et al., 2019). Several facial anomalies have been also described, such as short palpebral fissures, a thin upper lip, and a smooth philtrum (del Campo and Jones, 2017), among others (Wozniak et al., 2019).

Cognitive and behavioral deficits have been repeatedly identified in FASD patients as a consequence of both functional and anatomical brain abnormalities (Bjorkquist et al., 2010; Candelaria-Cook et al., 2021; Donald et al., 2016; Fryer et al., 2012; Lebel et al., 2008; Nardelli et al., 2011; Roussotte et al., 2012). Thus, beyond global intellectual deficits (reported in a high proportion of individuals with FASD) (Kautz-Turnbull and Petrenko, 2021; Popova et al., 2016), attention, memory, language, visuospatial skills, and executive functioning impairments have been consistently described in FASD (Hyland et al., 2023; Maya-Enero et al., 2021; Quan et al., 2018; Wozniak et al., 2019). At the behavioral level, FASD is associated with high levels of hyperactivity and impulsivity and low frustration tolerance (Bhatara et al., 2006), showing high irritability and other affective difficulties (e.g., low self-esteem) that impair social skills such as theory of mind (Maya-Enero et al., 2021; Quan et al., 2018; Sans-Fito et al., 2019; Wozniak et al., 2019).

It is important to note that FASD exhibits a very high rate of comorbidity with other neurodevelopmental conditions (Astley, 2010), such as the attention-deficit/hyperactivity disorder (ADHD) (O’Malley, 2007; Rasmussen et al., 2010), autism spectrum disorders (Clark et al., 2024), oppositional defiant disorder (Chasnoff et al., 2015), disruptive behavior disorders (Mela et al., 2013; Romero-González et al., 2020; Stevens et al., 2013), among other neurodevelopmental disorders (ND) (Astley et al., 2009; Clark et al., 2024; Weyrauch et al., 2017). This elevated comorbidity significantly complicates both accurate diagnosis and effective treatment (Wozniak et al., 2019). Indeed, at some point in their lives, most FASD patients have received multiple misdiagnoses before obtaining a correct identification of their condition of FASD (Chasnoff et al., 2015; Engesether et al., 2024). Differentiating FASD from other ND is particularly challenging, especially from ADHD, since a considerable number of individuals with FASD are initially diagnosed with these conditions (Engesether et al., 2024).

Some studies have pointed out the presence of syndrome-specific patterns that may guide diagnostic clarification. For example, executive functioning difficulties are consistently reported as more severe and widespread in FASD than in ADHD, especially in domains such as working memory, set-shifting, and planning (Mattson et al., 2013). A meta-analysis confirmed that although both groups show impairments in executive functioning, they were global and more pronounced in FASD (high effect size: *d* = 0.82) compared to ADHD (*d* = 0.55) (Khoury and Milligan, 2016). Broader neurocognitive reviews have similarly emphasized a core profile of deficits in learning, memory, language, social skills, and moral reasoning in FASD, suggesting that learning difficulties represent a central and pervasive feature of the syndrome, beyond the attentional impairments typically observed in ADHD (Nash, 2008). In this vein, findings on adaptive functioning in children with FASD reported significant deficits in communication, daily living skills, socialization and aggressive behaviors compared not only to typically developing peers (Jirikowic et al., 2008; Kodituwakku and Kodituwakku, 2014) but also to children with ADHD or other ND (Åse et al., 2012; Benson et al., 2023; Boseck et al., 2015; Coriale et al., 2013). Importantly, adaptive abilities in FASD follow a distinctive developmental trajectory. Thus, while children with learning disorders often show improvements in social functioning with age, those with FASD tend to experience a decline, reflecting a progressive vulnerability that accumulates across development (Åse et al., 2012; Thomas et al., 1998; Whaley et al., 2001). In summary, it has been proposed that FASD symptoms tend to be more complex and heterogeneous (with poorer overall functioning) than any of the comorbid conditions when evaluated in isolation (Mattson et al., 2019).

Despite the wide presence of FASD-related deficits, the neurocognitive, behavioral, and socio-emotional profile of FASD remains incompletely characterized (Maya-Enero et al., 2021). Previous studies addressing this issue have relied primarily on parent-reported questionnaires (Benson et al., 2023; Fagerlund et al., 2011; Nash et al., 2006; Olson and Sparrow, 2021), or on neuropsychological measures restricted to specific domains (Aragón et al., 2008b; Boseck et al., 2015; Khoury et al., 2015; Raldiris et al., 2018). Moreover, although large collaborative studies have implemented more extensive assessment batteries, these comparisons have typically been limited to FASD, ADHD and controls, without including children belonging to a broader range of ND (Lange et al., 2017; Mattson et al., 2013; Sakano et al., 2019).

Recognizing FASD syndrome-specific patterns is essential to be distinguished from typically developing peers, but especially from other ND as misclassification can result in inappropriate interventions that fail to address the complex and heterogeneous needs of individuals with FASD (Boseck et al., 2015; Lange et al., 2019). Therefore, the main objective of the present study was to comprehensively assess the neurocognitive, behavioral and socio-emotional profile of children with FASD using standardized protocols to identify both common and distinctive core features with respect to children diagnosed with other ND. Given the considerable clinical overlap between these neurodevelopmental conditions, this comprehensive comparative approach is expected to make a relevant contribution for improving diagnostic accuracy and understanding syndrome-specific trajectories.

## 2 Materials and methods

### 2.1 Participants

A total of 86 children participated in the study. The mean age was 11.00 years (SD = 2.30), with ages ranging from 6 to 15 years old. All participants were clinically referred for evaluation of suspected FASD or ND between 2018 and 2024. Following clinical assessment (see physical and Neuropsychological Assessment section), participants were assigned to one of two groups: (a) FASD group (*N* = 46) or (b) ND group (*N* = 30). We found no group differences in age [U = 538.5; *p* = 0.104]. Medians and interquartile range (IQR) for ND group were 10 (IQR = 2) and 12 (IQR = 5) for FASD group. All participants had normal or corrected-to-normal vision, no history of neurological disorders affecting cognitive function (e.g., stroke, brain tumors, epilepsy, or head trauma), and no history of psychosis, substance dependence, or substance abuse. Additionally, participants from adoptive backgrounds must have been adopted for at least 2 years and be fluent in Spanish. Sociodemographic data of both FASD and ND groups are detailed in Table 1.

**Table 1:** Means and standard deviations (in parenthesis) of sex, age, hand dominance and diagnostic category.

Variables	FASD group	ND group
*N*	46	30
Sex		
Male	26	10
Female	20	20
Age	11.54 (2.69)	10.50 (1.87)
Hand dominance		
Left-Handed	24	4
Right-Handed	22	26
Diagnostic		
FAS	11	–
pFAS	23	–
ARND	12	–
ARBD	0	–
ADHD	–	11
Dyslexia	–	9
ULD	–	10

FAS, fetal alcohol syndrome; pFAS, partial fetal alcohol syndrome; ARND, alcohol related neurodevelopmental disorders; ARBD, alcohol-related birth defects; ADHD, attention deficit/hyperactivity disorder; ULD, unspecified developmental disorder of scholastic skills.

#### 2.1.1 FASD group

Fifty-six patients were initially referred from the Spanish foundation of patients affected by fetal alcohol syndrome (www.visualteaf.com) and various neuropediatric services of public hospitals within the Community of Madrid (Spain). Patients were assessed according to the FASD diagnostic criteria established by the Institute of Medicine (Hoyme et al., 2016). Following the diagnostic evaluation (see Procedure section for details), 46 children (26 male, 20 female) fulfilled the diagnostic criteria outlined in the Hoyme et al. (2016) guidelines. Accordingly, 11 children were diagnosed with Fetal Alcohol Syndrome (FAS), 23 with Partial Fetal Alcohol Syndrome (pFAS) and 12 as Alcohol-Related Neurodevelopmental Disorder (ARND). None of the children met criteria for Alcohol-Related Birth Defects (ARBD). All patients in the FASD group had been adopted from the following countries of origin: Russia (73.9%), Spain (6.5%), Ukraine (4.3%), Romania (4.3%), Bolivia (2.2%), Poland (2.2%), Colombia (2.2%), India (2.2%) and Kazakhstan (2.2%). It is noteworthy that 10 patients with suspected FASD did not fulfill diagnostic criteria due to either absence of confirmed prenatal alcohol exposure or lack of phenotypic FASD features and were excluded from the study. Consequently, the final FASD sample consisted of forty-six participants who met the diagnostic FASD criteria.

#### 2.1.2 ND group

The ND cohort (*N* = 30) was referred primarily from neuropediatric services of public hospitals and from school-based evaluations within the Community of Madrid (Spain). Thus, the final ND contrast group comprised 30 participants (10 males, 20 females) diagnosed with specific ND according to the ICD-10 (Second edition, World Health Organization, 2009). Accordingly, 12 children were diagnosed with ADHD (F90.0), 9 with Dyslexia (F81.0), and 19 with Unspecified Developmental Disorder of Scholastic Skills (ULD) (F81.9). Children belonging to the ND group underwent equivalent neuropsychological assessments as those of children suspected of having FASD (see Neuropsychological Assessment section). Inclusion criteria for the ND group were as follows: (a) no having documented history of maternal alcohol use during pregnancy in medical records (e.g., maternal self-report, child welfare records, or official legal documentation); (b) absence of phenotypic or clinical features suggestive of prenatal alcohol exposure; and (c) normal somatic growth parameters according to standardized growth charts.

### 2.2 Procedure

Participants and legal custodians gave written clinical informed consent for their involvement. The study was approved by the Rey Juan Carlos University Research Ethics Board (Protocol: 25052020212520) and followed all the requirements given by this committee.

The procedure consisted of seven sessions spread over a period of 2 weeks. The initial session was devoted to morphological and somatometric examination performed by a single experienced dysmorphologist (duration of 30 min). The remaining six sessions, each lasting around 30 min, involved a comprehensive neuropsychological assessment conducted by a single trained neuropsychologist.

#### 2.2.1 Somatometric assessment

The somatometric evaluation focused on identifying the characteristic features and growth patterns commonly associated with FASD. The assessment included measurement of height, weight, and head circumference, which were compared with age- and sex-specific normative values to detect possible growth restriction or microcephaly (Pc < 10).

In addition, a detailed facial examination was performed. This involved systematic observation of key dysmorphic features, including short palpebral fissures, smooth philtrum, and thin upper lip, as well as any other minor anomalies. Validated scoring systems were used to ensure consistency across raters (Hoyme et al., 2016). The examination also screened congenital malformations or structural anomalies in other systems (e.g., cardiac, skeletal, neurological signs) that may occur in association with FASD.

#### 2.2.2 Neuropsychological assessment

Children completed a battery of standardized neuropsychological tests. Intellectual functioning was assessed using the Spanish adaptation of the *Wechsler Intelligence Scale for Children (WISC-V)* (Wechsler, 2005). This instrument is designed to measure overall intellectual ability (IQ) through the full-scale. In addition, its structure -comprising 15 subtests- permits a detailed evaluation of specific cognitive domains, including indices of Verbal Comprehension (VCI), Visual Spatial (VSI), Fluid Reasoning (FRI; including mathematical reasoning), Working Memory (WMI; both verbal and visual), and Processing Speed (PSI) skills. Furthermore, the *WISC-V* provides five secondary index scores that enable a fine-grained characterization of cognitive functioning: Quantitative Reasoning Index (QRI), Auditory Working Memory Index (AWMI), Nonverbal Index (NVI), General Ability Index (GAI), and Cognitive Proficiency Index (CPI). The Spanish adaptation of the *WISC-V* has demonstrated high internal consistency (*α* = 0.88–0.93).

Attentional functioning was evaluated using the Spanish adaptation of the *d2 Test of Attention* (Brickenkamp, 2002, 2012), and the *Stroop Test* (Golden, 2005; Stroop, 1992). The *d2 Test* assesses sustained attention and processing speed, while the *Stroop Test* allows measuring selective attention and inhibitory control (also involving sustained attention and processing speed). The *d2 Test* consists of 650 stimuli arranged in 14 lines, where participants are required to identify a specific target stimulus (the letter “d” with two quotation marks) as quickly and accurately as possible while ignoring distractors. The *Stroop Test* includes three experimental conditions: (a) word reading (processing speed), (b) color naming (processing speed), and (c) color-word interference (inhibitory control). Psychometric properties indicate adequate reliability for all measures: the *d2 Test* (*α* = 0.90) and the *Stroop Test* (α = 0.75). Collectively, these instruments are widely recognized as valid and reliable tools for the assessment of attentional performance.

Memory functioning was assessed using the *Child Verbal Learning España-Complutense Test (TAVECI)* (Benedet Álvarez et al., 2001). *TAVECI* is designed to evaluate verbal memory through multiple word lists, allowing the assessment of free recall, cued recall, and recognition processes in both short and long term memory. In addition, the *Test of Memory and Learning (TOMAL)* (Reynolds et al., 2012) was also administered. The *TOMAL* consists of 14 subtests that provide indices related to verbal memory (VMI), non-verbal memory (NVMI), composite memory (CMI), and delayed recall (DRI) (Thaler et al., 2010). Both instruments have demonstrated adequate psychometric properties, with high internal consistency coefficients: TAVECI (α = 0.86) and TOMAL (α = 0.80).

Executive functioning was assessed using the *Neuropsychological Assessment of Executive Functions in Children (ENFEN)* (Portellano et al., 2009). The ENFEN battery evaluates four core executive domains: verbal fluency, sustained and alternating attention, planning, and inhibition. Specifically, it comprises several subtests: (a) phonemic and semantic verbal fluency (executive verbal production); (b) trails task (scanning, sustained and alternating attention, cognitive flexibility); (c) construction of the gray paths (planning and organization); and (d) resistance to interference (inhibitory control). Psychometric evidence supports the reliability of the instrument, with high internal consistency (α = 0.70–0.85).

Finally, socio-emotional symptoms were assessed through the *Child Behavior Checklist (CBCL/6–18)* (García et al., 1997). The test has 112 questions that are used to evaluate affective and behavioral problems (anxiety, depression, social problems, thought problems, attention deficits, and aggressive behavior) in children. This test was completed by the patient’s parents or legal guardians. *CBCL* has excellent psychometric properties (Albores-Gallo et al., 2007), and have been widely used in Spanish population (Portillo-Reyes et al., 2016).

### 2.3 Data analysis

Neuropsychological scores in both the FASD and ND groups were examined according to the normative ranges specified for each test. Normative scores from the primary and secondary indexes of the *WISC-V* and *TOMAL* batteries were used, along with centile scores from the *d2* and *ENFEN* test. Moreover, standardized typical scores from the *Stroop Test*, *TAVECI* and *CBCL/6–18* were considered.

Given that the distribution of most neuropsychological variables did not meet the assumption of normality (Shapiro–Wilk test, *p* < 0.05), non-parametric tests were applied. Specifically, group differences (FASD vs. ND) were analyzed using the Mann–Whitney U test for independent samples. Effect sizes were calculated using the rank-biserial correlation (rₛ). Because multiple variables were tested, the family-wise error rate (FWER) was controlled using the Holm–Bonferroni sequential correction procedure (Holm, 1979). Statistical significance was interpreted based on adjusted *p*-values.

An additional complementary analysis was conducted using IQ-matched subsamples (85–115). This comparison was performed following the same statistical procedures as the main analysis, and the detailed are provided in Supplementary material. All statistical analyses were conducted using SPSS (version 29.0; SPSS Inc., Chicago, IL, USA).

## 3 Results

### 3.1 Analyses of normative data

As compared to normative ranges, the global cognitive assessment (*M* = 100; SD = 15) revealed clinically deficits in the FASD group, reflected by markedly low IQ scores. These impairments were particularly evident in verbal comprehension, working memory, and fluid reasoning indices, whereas the ND group consistently performed within the normative range. Consistent with this pattern, mean performance of the FASD group was below average levels across attentional (*M* = 50; SD = 10), memory (*M* = 100; SD = 15; *M* = 0; SD = 1), and executive domains (*M* = 50; SD = 10), including accuracy, commission and omission errors, processing speed sustained and alternating attention, verbal memory, planning, and inhibition inhibitory control. Means and standard deviations (in parenthesis) of each cognitive measure associated with each group (FASD and ND) are shown in Table 2. By contrast, the ND group performed within average normative ranges except for *d2* Test where children showed a rate of commission errors above average.

**Table 2:** Means and standard deviations (in parenthesis) of each cognitive index by group (FASD and ND).

Variables	FASD	ND	Mann–Whitney U
Global cognition			
WISC-V			
Full-Scale (IQ)	78.39 (12.37)	97.17 (10.69)	U = 172.5, *p* < 0.001, r_s_ = 0,63*
Verbal comprehension (VCI)	80.74 (15.36)	97.37 (10.86)	U = 248.5, *p* < 0.001, r_s_ = 0,54*
Visual spatial (VSI)	87.78 (13.74)	101.12 (11.19)	U = 314.5, *p* < 0.001, r_s_ = 0,46*
Fluid reasoning (FRI)	83.22 (11.48)	99.12 (10.96)	U = 197.0, *p* < 0.001, r_s_ = 0,60*
Working memory (WMI)	82.91 (14.49)	97.73 (10.60)	U = 294.5, *p* < 0.001, r_s_ = 0,48*
Processing speed (PSI)	87.91 (15.37)	102.20 (15.89)	U = 371.5, *p* = 0.005, r_s_ = 0,39*
Quantitative reasoning (QRI)	79.13 (11.88)	97.63 (12.09)	U = 182.0, *p* < 0.001, r_s_ = 0,62*
Auditory working memory (AWMI)	81.91 (16.06)	98.23 (9.30)	U = 236.5, *p* < 0.001, r_s_ = 0,55*
Nonverbal Processing (NVI)	82.09 (13.32)	98.60 (11.22)	U = 247.5, *p* < 0.001, r_s_ = 0,54*
General ability (GAI)	80.50 (11.92)	97.83 (10.61)	U = 191.0, *p* < 0.001, r_s_ = 0,61*
Cognitive proficiency (CPI)	82.83 (15.95)	100.27 (13.39)	U = 294.5, *p* < 0.001, r_s_ = 0,48*
Attention			
D2			
Precision (accuracy)	24.28 (24.71)	52.23 (27.00)	U = 288.0, *p* < 0.001, r_s_ = 0,49*
Commission errors	18.76 (21.19)	34.43 (25.61)	U = 411.5, *p* = 0.014, r_s_ = 0,34*
Omission errors	26.15 (25.38)	41.50 (28.27)	U = 449.5, *p* = 0.020, r_s_ = 0,29*
Stroop			
Words reading	33.74 (11.91)	41.73 (7.32)	U = 400.0, *p* = 0.014, r_s_ = 0,35*
Color naming	34.89 (10.55)	44.23 (7.51)	U = 322.0, *p* < 0.001, r_s_ = 0,45*
WC interference	34.78 (9.36)	42.20 (6.54)	U = 353.5, *p* = 0.003, r_s_ = 0,41*
Memory			
TOMAL			
Verbal Memory Index (VMI)	77.49 (12.37)	96.03 (12.35)	U = 185.0, *p* < 0.001, r_s_ = 0,61*
Non-verbal Memory Index (NVMI)	83.84 (12.86)	96.50 (11.57)	U = 302.5, *p* < 0.001, r_s_ = 0,47*
Composite Memory Index (CMI)	79.71 (11.57)	95.73 (10.91)	U = 194.5, *p* < 0.001, r_s_ = 0,60*
Delay Recall Index (DRI)	85.71 (11.38)	99.40 (9.46)	U = 217.0, *p* < 0.001, r_s_ = 0,57*
TAVECI			
Immediate free recall (IFR)	−1.57 (1.38)	−0.31 (0.94)	U = 405.0, *p* = 0.012, r_s_ = 0,35*
Delayed free recall (DFR)	−1.53 (1.44)	−0.12 (0.86)	U = 287.0, *p* < 0.001, r_s_ = 0,50*
Cued recall (CR)	−1.32 (1.09)	−0.08 (0.95)	U = 264.0, *p* < 0.001, r_s_ = 0,52*
Recognition Recall (RR)	−0.93 (1.41)	−0.18 (1.29)	U = 424.0, *p* = 0.014, r_s_ = 0,032*
Executive functions			
ENFEN			
Visual scanning	23.65 (17.59)	44.47 (17.83)	U = 279.0, *p* = 0.001, r_s_ = 0,51*
Flexibility	21.96 (18.08)	37.17 (18.29)	U = 348.5, *p* < 0.001, r_s_ = 0,43*
Phonemic fluency	61.30 (31.52)	67.00 (24.93)	U = 622.5, *p* = 0.469, r_s_ = 0.00
Semantic fluency	53.48 (28.69)	70.67 (20.50)	U = 450.0, *p* = 0.030, r_s_ = 0,29*
Planning solving	16.89 (13.11)	43.32 (18.25)	U = 197.0, p < 0.001, r_s_ = 0,64*

Mann–Whitney U tests of neuropsychological measures are shown. Statistically significant results are marked with an asterisk, and their size effects are also reported.

Finally, regarding the assessment of behavioral and socio-emotional symptoms through *CBCL/6–18* inventory, the FASD group obtained scores above normative average only in attention problems domain (M > 70). Considering the rest of domains both children’s groups, FASD and ND, exhibited levels of symptoms (anxiety, depression, social problem, attention deficits, and aggressive behavior) below clinical ranges (see Table 3).

**Table 3:** Means and standard deviations (in parenthesis) of each socio-emotional behavior index (of CBCL/6–18 test) by group (FASD and ND).

Variables	FASD	ND	Mann–Whitney U
Anxiety	65.87 (6.29)	59.53 (6.07)	U = 1046.0, *p* < 0.001, r_s_ = 0,44*
Depression	61.26 (8.22)	59.70 (10.1)	U = 803.0, *p* = 0.229, r_s_ = 0,14
Somatic complains	60.09 (6.84)	58.32 (8.09)	U = 822.0, *p* = 0.318, r_s_ = 0,16
Social problems	68.79 (7.954)	61.12 (8.73)	U = 1065.0, *p* < 0.001, r_s_ = 0,46*
Thought problems	63.98 (8.85)	56.27 (5.34)	U = 1041.0, *p* < 0.001, r_s_ = 0,43*
Attention problems	71.63 (6.92)	65.07 (7.34)	U = 1012.5, *p* = 0.002, r_s_ = 0.39*
Rule-breaking behavior	60.48 (7.59)	56.07 (4.32)	U = 905.5, *p* = 0.06, r_s_ = 0,26
Aggressive behavior	65.07 (9.10)	56.77 (5.88)	U = 1061.5, *p* < 0.001, r_s_ = 0,45*

Mann–Whitney U tests of neuropsychological measures are shown. Statistically significant results are marked with an asterisk, and their size effects are also reported.

### 3.2 Analyses on neuropsychological functions

#### 3.2.1 Global cognitive status

**Figure 1:**
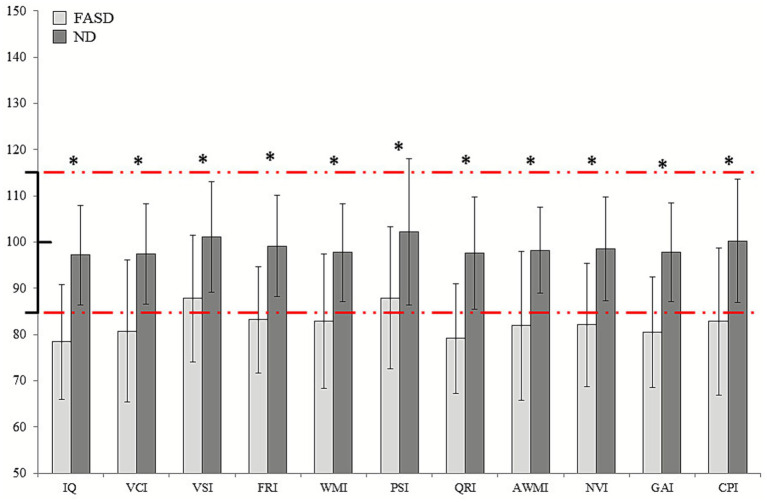
Group differences in neuropsychological measures of global cognitive status *(WISC-V)*. Means and standard deviations are shown for FASD patients and ND, with significant results marked with an asterisk. Normative ranges are marked in red lines.

Mann–Whitney U tests revealed significant statistical differences between the FASD and ND groups in every *WISC-V* index (Figure 1). In specific, FASD patients showed lower scores in IQ, Verbal Comprehension, Visual Spatial, Fluid Reasoning, Working Memory, Processing Speed, Quantitative Reasoning, Auditory Working Memory, Nonverbal Processing, General Ability, and Cognitive Proficiency compared to the ND group (*p* < 0.01). Full statistical results related to the principal and secondary *WISC-V* indexes can be found in Table 2.

#### 3.2.2 Attention

**Figure 2:**
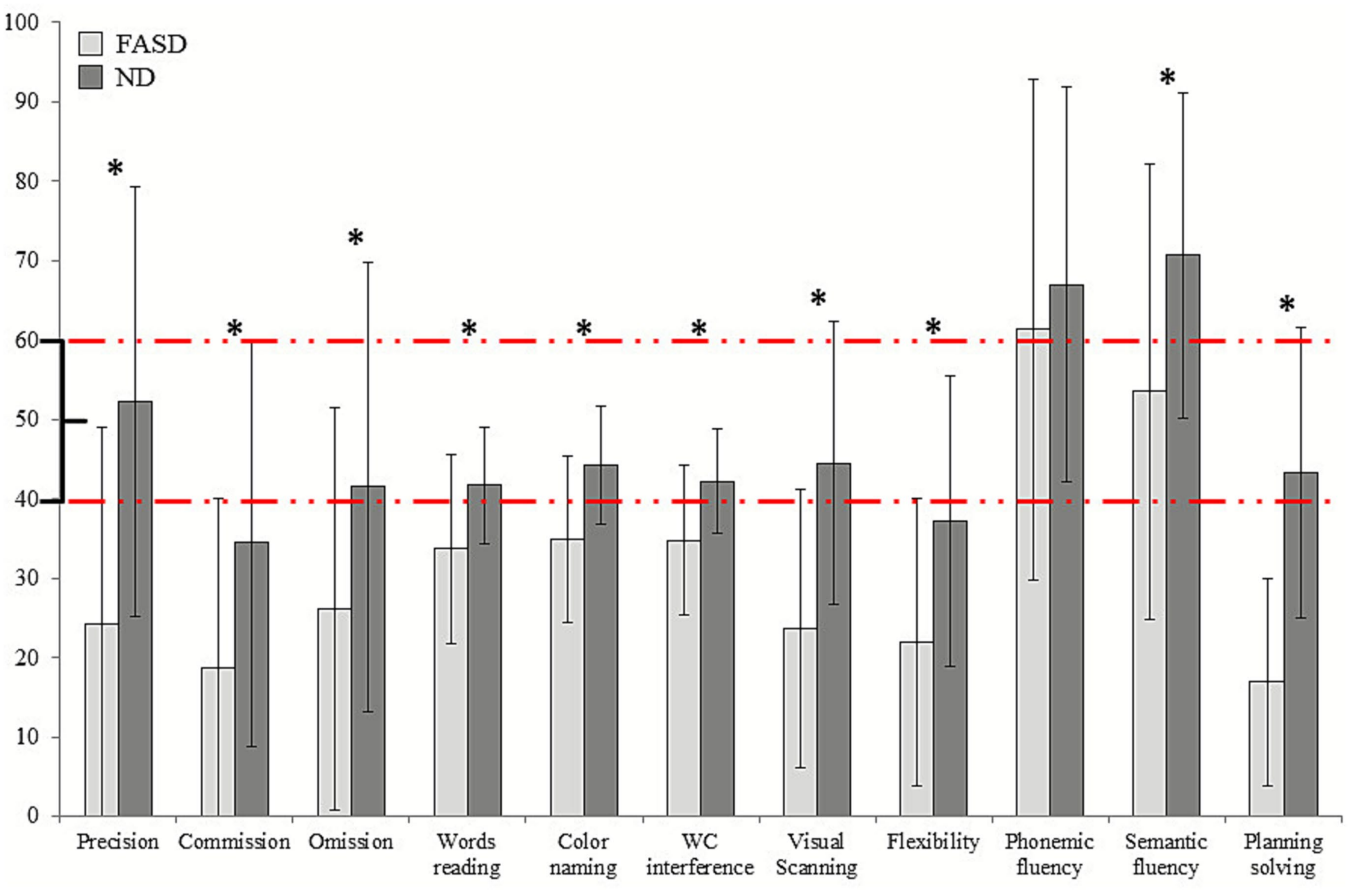
Group differences in neuropsychological measures of attention *(d2 and Stroop Test)* and executive functions *(ENFEN)*. Means and standard deviations are shown for FASD patients and ND, with significant results marked with an asterisk. Normative ranges are marked in red lines.

Mann–Whitney U tests revealed significant group differences between ND and FASD children on several attentional indices (Figure 2). FASD group obtained significantly lower scores in the main outcomes of the *d2 Test*, including Accuracy (*p* < 0.001), Commission errors (*p* = 0.014), and Omission errors (*p* = 0.020). Similarly, in the *Stroop Test*, the FASD group exhibited significantly poorer performance across specific measures, with lower scores in Word reading (*p* = 0.014), Color naming (*p* < 0.001) and Color–Word interference (*p* = 0.003). Full statistical results can be found in Table 2.

#### 3.2.3 Memory

**Figure 3:**
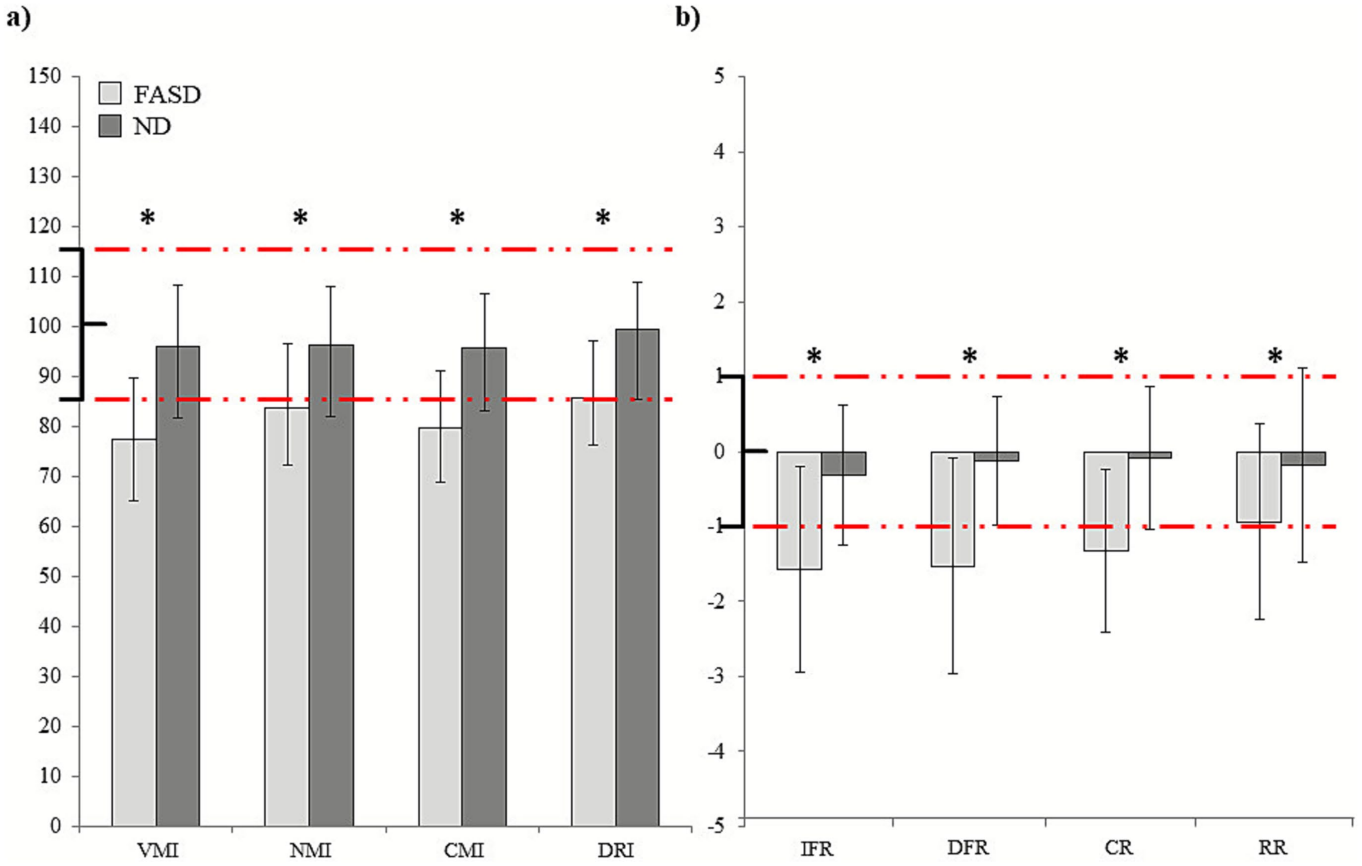
Measures of memory. Group differences in neuropsychological measures of memory: **(a)** TOMAL and **(b)** TAVECI. Means and standard deviations are shown for FASD patients and ND, with significant results marked with an asterisk. Normative ranges are marked in red lines.

Mann–Whitney U tests yielded significant differences between groups for all *TOMAL* indices (see Figure 3). Children with FASD performed significantly lower in Verbal Memory, Non-verbal Memory, Composite Memory, and Delay Recall than the ND group (all *p* < 0.001). Similarly, in the TAVECI test, the FASD group showed significantly lower performance for immediate free recall, delayed free recall, recognition recall accuracy and cued delayed recall as compared to ND children (all *p* < 0.05).

#### 3.2.4 Executive functions

Significant group differences were found (FASD and ND) in all outcomes provided by *ENFEN* (Figure 2). Once again, FASD children exhibited significantly lower scores in Semantic fluency, Visual Scanning, Cognitive Flexibility (trial 1 and 2, respectively) and Planning solving than the ND group (all *p* < 0.05). In contrast, no significant differences emerged for Phonemic fluency between groups (*p* = 0.469).

### 3.3 Analyses on socio-emotional behavior

**Figure 4:**
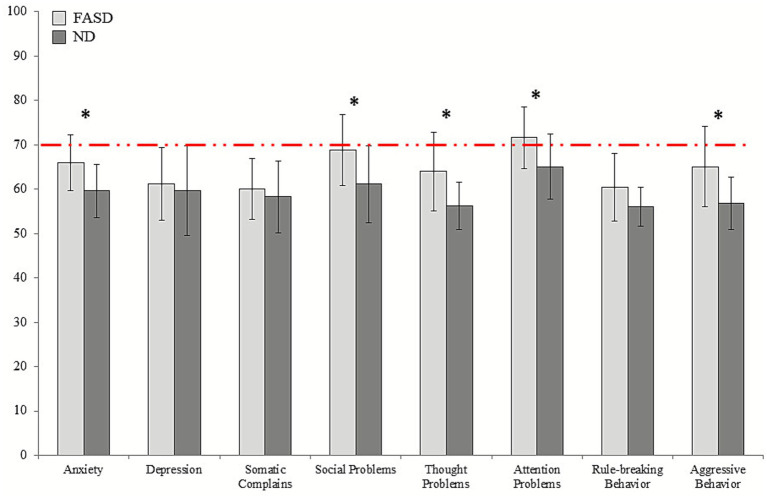
Group differences in socio-emotional behavior measures *(CBCL/6–18)*. Means and standard deviations are shown for FASD patients and ND, with significant results marked with an asterisk. Clinical normative ranges are marked in red lines.

Analyses revealed significant group differences on several *CBCL/6–18* scales between FASD and ND groups (Figure 4). The FASD group showed significantly higher scores in Anxiety, Social Problems, Thought Problems, Attention Problems, and Aggressive Behavior (all *p* < 0.05). In contrast, we found no significant group differences for Depression (*p* = 0.229), Somatic Complaints (*p* = 0.159) and Rule-Breaking Behavior (*p* = 0.06). Full statistical results can be found in Table 3.

### 3.4 IQ-matched analysis

A complementary analysis restricted to IQ-matched subsamples (85–115) yielded the same pattern of significant differences between groups; detailed results are available in Supplementary Tables S1, S2.

## 4 Discussion

The present study aimed to delineate the specific and differentiated profile of children with FASD in both the cognitive and socio-emotional spheres. Our findings indicate that FASD children experience a marked pattern of cognitive deficits compared to those diagnosed with ND, mainly characterized by lower global intellectual functioning and a pronounced impairment in attention, memory and executive functions. Although scores derived from parent reported questionnaires showed that children with FASD significantly differed from ND group (worse outcomes in some behavioral and socio-emotional domains), the absence of clinically significant impairments in FASD suggest that their adaptive behavioral functioning remains generally preserved despite marked cognitive weaknesses.

Present findings converge with previous literature showing that FASD is characterized by a widespread, yet uneven, neurocognitive impairment (Aragón et al., 2008a; Maya-Enero et al., 2021). Previous evidence argued that FASD show disproportionately greater deficits in complex cognitive tasks (e.g., planning, logical memory, verbal fluency) than in simpler ones (Aragón et al., 2008b; Khoury and Milligan, 2016). However, the performance observed in FASD children reflects lower scores in most of *WISC-V* indexes compared to ND children, as previously reported (Kodituwakku and Kodituwakku, 2014). Similarly, deficits extended to attentional measures, where children with FASD performed poorly on sustained and selective attention tasks, as well as to verbal and non-verbal memory indices, with especially marked difficulties in free recall. Such a pattern has led several authors to argue that FASD presents a distinctive neurocognitive phenotype, although a universally accepted profile is still debated (Lange et al., 2017; Nash, 2008).

The profile exhibited by FASD children suggests the presence of *quantitative* impairments (i.e., a lower IQ), but also qualitative, reflecting inefficiencies in information processing, slower cognitive tempo, and marked difficulties when tasks demand high-order functions such as planning, working memory, or cognitive flexibility (Aragón et al., 2008a; Kodituwakku and Kodituwakku, 2014). Nevertheless, the difficulty in establishing a consensual profile stems from the heterogeneity of manifestations across individuals, the variability in exposure patterns, and the frequent overlap with other neurodevelopmental conditions (Lange et al., 2017; Mattson et al., 2019). Despite of it, converging evidence suggests that the impairments observed in FASD are not randomly distributed, but instead cluster in a way that reflects the underlying neurobiological impact of prenatal alcohol exposure (Khoury and Milligan, 2016). When comparing with other ND conditions, both overlaps and specificities emerge. For example, some authors have pointed out that, although attention difficulties in FASD may resemble those observed in ADHD, their qualitative nature appears to be different; rather than hyperactivity or impulsivity, the main problem seems to lie in poor executive self-regulation, which manifests itself in difficulties in maintaining attention, organizing information and adapting to changing task demands (Nash, 2008; Raldiris et al., 2018). In this line, although learning disabilities are also frequent, while striking weaknesses in visuospatial reasoning, processing speed, and flexible problem solving are detected in FASD children, verbal skills are relatively preserved (Aragón et al., 2008a; Benson et al., 2023; Thomas et al., 1998; Whaley et al., 2001). It provides a neuropsychological signature that distinguishes FASD from other ND.

At the socio-emotional level, parental reports revealed that children with FASD scored higher than their peers with ND on anxiety, social and thought problems, attentional difficulties, and aggression. It is important to note that high scores described in the FASD group largely remained within normative ranges in contrast with the pronounced cognitive impairment shown across cognitive domains. In this sense, clinical studies have documented that inattention is a more reliable marker of FASD than hyperactivity/impulsivity (Aragón et al., 2008a). Thus, behavioral difficulties in FASD tend to cluster around challenges in emotional regulation, executive functions, and adaptive functioning, yet their severity varies widely depending on contextual stressors such as unstable caregiving, educational exclusion, or lack of appropriate interventions (McDougall et al., 2020). Comparative research further highlights that, although these difficulties often overlap with those observed in ADHD, oppositional defiant disorder, or autism spectrum conditions, their qualitative nature appears distinct, with regulation problems more tightly linked to executive dysfunction than to hyperactivity or impulsivity (Carrick and Hamilton, 2023; O’Malley and Nanson, 2002). This distinction has clinical relevance, as interventions designed for ADHD may not adequately address the specific regulatory deficits and adaptive challenges in FASD (Raldiris et al., 2018).

Taken together, these findings support the view that behavioral difficulties in FASD are not inevitable or uniformly severe, but contingent outcomes shaped by the interplay of primary neurocognitive impairments and environmental factors. This developmental-ecological perspective underscores the importance of proactive identification, family-centered supports, and educational strategies to sustain functioning within normative ranges and to prevent escalation toward secondary disabilities such as school failure, legal involvement, or mental health problems -outcomes that are largely preventable through early and sustained intervention- (Graham, 2014; Patrenko et al., 2014; Roozen et al., 2018). Contemporary perspectives further emphasize the value of moving beyond a deficit-only approach to adopt strength-based and quality-of-life frameworks, which may reduce stigma and foster resilience (Olson and Sparrow, 2021). Structured educational strategies, consistent caregiving environments, and family-centered therapies have been highlighted as particularly effective in promoting stability and compensating for core deficits (Kalberg and Buckley, 2007), while more recent multidisciplinary neurorehabilitation programs have reported promising outcomes, including gains in self-regulation, attention, and adaptive functioning (Fernandes-Magalhaes et al., 2023).

There is still ongoing debate as to whether socio-emotional impairments and behavioral difficulties should be regarded as a core feature of the syndrome, linked to primary deficits in higher-order processing (Lange et al., 2018; Nash et al., 2006; Sakano et al., 2019), or whether they are better conceptualized as secondary outcomes shaped by the interaction between neurocognitive vulnerabilities and contextual factors, including delayed diagnosis, lack of intervention, and limited environmental support, as suggested by evidence of heterogeneity across clinical and cohort studies (Benson et al., 2023; Boseck et al., 2015; Olson and Sparrow, 2021; Reid et al., 2017). Indeed, growing literature supports the notion that behavioral manifestations in FASD are better conceptualized as secondary rather than primary symptoms of the disorder (Coriale et al., 2013; Streissguth et al., 1996).

Several limitations of the present study should be acknowledged. First, although groups were carefully matched, potential unmeasured confounding variables, such as differences in early caregiving environments, prenatal exposures beyond alcohol, or comorbid neurodevelopmental conditions, could have influenced the results. This limitation arises from the retrospective nature of data collection and the variability in documentation across different referral sources, which is common in clinical studies involving FASD patients. Second, the cross-sectional design precludes inferences regarding developmental trajectories, making it difficult to determine how cognitive and behavioral profiles evolve over time. Future research should employ larger and more diverse samples using longitudinal designs to track developmental changes in cognition and behavior across childhood and adolescence. Given the heterogeneity within FASD, person-centered analytic approaches and machine learning-based multivariate pattern analyses may prove useful for identifying neural biomarkers, delineating subgroups, and tailoring individualized interventions (Dyląg et al., 2021; Fernandes-Magalhaes et al., 2023; Ramos-Triguero et al., 2024).

In summary, the distinctive profile observed in FASD, characterized by generalized reductions in overall intellectual functioning along with disproportionately greater impairments in specific neurocognitive domains such as attention, memory, and executive functions, provides a consistent basis for differentiating FASD from other NDs. Whereas other developmental disorders often show more circumscribed or domain-limited difficulties, the convergence of broad cognitive weakness and marked domain-specific deficits appears particularly characteristic of prenatal alcohol exposure. Finally, behavior and daily functioning of children with FASD appear to be largely determined by contextual factors. Understanding this pattern strengthens the interpretation of FASD-related neurobehavioral outcomes and reinforces the need for early identification and targeted, evidence-based interventions to mitigate long-term adaptive challenges.

## Data Availability

The raw data supporting the conclusions of this article will be made available by the authors, without undue reservation.
