# Supplementary material for: Differentiating Fetal Alcohol Spectrum Disorder from other neurodevelopmental disorders: neurocognitive and socio-emotional evidence
Source: Front Neurosci. 2025 Dec 5;19:1716494. doi: 10.3389/fnins.2025.1716494 (PMC12714968; doi:10.3389/fnins.2025.1716494)
Supplement: Supplementary file 1 [file Data_Sheet_1.docx]

**Supplementary materials for “Differentiating Fetal Alcohol Spectrum Disorder (FASD) from other Neurodevelopmental Disorders: Neurocognitive and Socio-Emotional Evidence”**

Roberto Fernandes-Magalhaes, Lorena Labrado, David Ferrera, Marisa Fernández-Sánchez, Ana Belén del Pino, Dino Soldic, Sarai Pazos-García, María Eugenia De Lahoz, Irene Peláez, Paloma Barjola, Francisco Mercado

Analysis in IQ-Matched Subgroups

This supplementary analysis was conducted to determine whether the neurocognitive and socio-emotional differences observed between the FASD and ND groups persisted after controlling for general intellectual functioning. To this end, only participants whose Full-Scale IQ fell within the normal range (i.e., 85-115) were included, resulting in a subsample of 15 children with FASD and 23 children with ND.

| **Variables** | **FASD** | **ND** | **Mann–Whitney U** |
| --- | --- | --- | --- |
| Anxiety | 64.67 (7.22) | 58.30 (5.92) | U = 85.50, p = 0.008, rs = 0,50* |
| Depression | 62.67 (8.92) | 60.35 (11.03) | U = 136.5, p = 0.286, rs = 0,20 |
| Somatic Complains | 58.40 (6.63) | 57.96 (7.98) | U = 158.0, p = 0.680, rs = 0,08 |
| Social Problems | 65.63 (8.75) | 61.47 (9.36) | U = 125.0, p = 0.162, rs = 0,27 |
| Thought Problems | 60.73 (9.26) | 55.61 (5.63) | U = 117.0, p = 0.101, rs = 0,32 |
| Attention Problems | 69.53 (7.64) | 64.87 (6.55) | U = 111.0, p = 0.068, rs = 0,35 |
| Rule-breaking Behavior | 60.40 (7.44) | 56.04 (4.80) | U = 117.0, p = 0.101, rs = 0,32 |
| Aggressive Behavior | 65.53 (9.03) | 57.00 (5.36) | U = 71.00, p = 0.002, rs = 0,58* |

Group comparisons were performed using the Mann–Whitney U test, following the same statistical procedures applied in the primary analysis. The results demonstrated that the same pattern of significant differences was maintained. In particular, the FASD group continuing to exhibit significantly lower performance across domains than children with ND. A summary of the statistical results for the IQ-matched subsample is presented in Supplementary Tables S1 and S2.

**Table S1.** Means and standard deviations (in parenthesis) of each socio-emotional behavior index (of CBCL/6-18 test in the IQ-matched subsamples (FASD and ND). Mann–Whitney U tests of neuropsychological measures are shown. Statistically significant results are marked with an asterisk, and their size effects are also reported.

| **Variables** | **FASD** | **ND** | **Mann–Whitney U** |
| --- | --- | --- | --- |
| **GLOBAL COGNITION** |  |  |  |
| **WISC-V** |  |  |  |
| Full-Scale (IQ) | 91.12 (5.19) | 99.04 (6.29) | U = 61.50, p < 0.001, rs = 0,64* |
| Verbal Comprehension (VCI) | 92.67 (11.28) | 97.74 (8.57) | U = 108.0, p = 0.050, rs = 0,37* |
| Visual Spatial (VSI) | 98.93 (10.10) | 102.04 (10.11) | U = 140.5, p = 0.344, rs = 0,18 |
| Fluid Reasoning (FRI) | 91.08 (10.74) | 100.70 (8.87) | U = 84.00, p = 0.007, rs = 0,51* |
| Working Memory (WMI) | 95.60 (10.16) | 100.43 (9.24) | U = 132.5, p = 0.235, rs = 0,23 |
| Processing Speed (PSI) | 97.27 (12.58) | 105.26 (15.20) | U = 120.0, p = 0.121, rs = 0,30 |
| Quantitative Reasoning (QRI) | 87.40 (9.38) | 99.48 (11.20) | U = 67.00, p = 0.001, rs = 0,61* |
| Auditory Working Memory (AWMI) | 92.53 (12.91) | 100.12 (7.53) | U = 79.00, p = 0.004, rs = 0,54* |
| Nonverbal Processing (NVI) | 95.27 (6.90) | 100.65 (8.63) | U = 113.5, p = 0.078, rs = 0,34 |
| General Ability (GAI) | 92.07 (8.25) | 99.22 (6.48) | U = 87.00, p = 0.010, rs = 0.49* |
| Cognitive Proficiency (CPI) | 96.93 (8.49) | 103.78 (11.63) | U = 118.5, p = 0.107, rs = 0,31 |
| **ATENTION** |  |  |  |
| **D2** |  |  |  |
| Precision (accuracy) | 31.20 (24.83) | 54.43 (29.86) | U = 90.50, p = 0.013, rs = 0,47* |
| Commission errors | 26.53 (26.41) | 32.26 (28.02) | U = 148.0, p = 0.478, rs = 0,15 |
| Omission errors | 25.40 (20.20) | 43.48 (31.40) | U = 112.5, p = 0.073, rs = 0,34 |
| **STROOP** |  |  |  |
| Words reading | 37.32 (8.83) | 41.91 (7.75) | U = 114.5, p = 0.083, rs = 0,32 |
| Color naming | 40.12 (7.15) | 44.39 (7.46) | U = 113.5, p =078, rs = 0,34 |
| WC interference | 37.87 (6.82) | 42.87 (6.37) | U = 99.00, p = 0.030, rs = 0,42* |
| **MEMORY** |  |  |  |
| **TOMAL** |  |  |  |
| Verbal Memory Index (VMI) | 83.93 (7.79) | 96.30 (13.29) | U = 79.00, p = 0.004, rs = 0,54* |
| Non-verbal Memory Index (NVMI) | 87.80 (9.68) | 98.30 (11.93) | U = 81.50, p = 0.006, rs = 0,52* |
| Composite Memory Index (CMI) | 84.93 (7.22) | 96.48 (11.69) | U = 63.50, p = 0.001, rs = 0,63* |
| Delay Recall Index (DRI) | 89.32 (10.1) | 98.57 (10.04) | U = 83.00, p = 0.007, rs = 0,51* |
| **TAVECI** |  |  |  |
| Immediate Free Recall (IFR) | -0.76 (0.84) | -0.47 (0.95) | U = 141.0, p = 0.359, rs = 0,18 |
| Delayed Free Recall (DFR) | -1.23 (1.14) | -0.32 (1.00) | U = 94.00, p = 0.018, rs = 0,45* |
| Cued Recall (CR) | -1.20 (1.19) | -0.26 (0.99) | U = 92.00, p = 0.016, rs = 0,46* |
| Recognition Recall (RR) | -1.23 (1.68) | -0.37 (1.43) | U = 118.5, p = 0.107, rs = 0,31 |
| **EXECUTIVE FUNCTIONS** |  |  |  |
| **ENFEN** |  |  |  |
| Visual Scanning | 32.73 (21.01) | 43.78 (19.27) | U = 121.5, p = 0.129, rs = 0,29 |
| Flexibility | 28.73 (15.57) | 37.57 (18.02) | U = 121.5, p = 0.129, rs = 0,29 |
| Phonemic fluency | 78.00 (25.41) | 64.35 (25.73) | U = 117.5, p = 0.101, rs = 0,31 |
| Semantic fluency | 66.67 (30.15) | 67.39 (19.82) | U = 161.0, p = 0.746, rs = 0,06 |
| Planning solving | 22.67 (15.32) | 43.91 (7.75) | U = 66.50, p = 0.001, rs = 0,61* |

**Table S2.** Means and standard deviations (in parenthesis) of each cognitive index in the IQ-matched subsamples (FASD and ND). Mann–Whitney U tests of neuropsychological measures are shown. Statistically significant results are marked with an asterisk, and their size effects are also reported.
